# Telomere Dysfunction as an Initiator of Inflammation: Clues to an Age-Old Mystery

**Published:** 2021-02-17

**Authors:** Deepavali Chakravarti, Ronald A. DePinho

**Affiliations:** Department of Cancer Biology, The University of Texas, MD Anderson Cancer Center, Houston, TX 77030, USA

**Keywords:** Telomere, Pathogenic, Inflammation

## Abstract

Inflammatory Bowel Disease (IBD) is a challenging medical condition that is driven by various genetic and environmental factors. Therapeutic opportunities for this disease remain limited due to the lack of in-depth understanding of the pathogenetic mechanisms and actionable targets driving the disease. Analysis of telomere dysfunctional mice and patients with genetic defects in telomere maintenance unexpectedly revealed phenotypes mirroring those observed in IBD. Molecular characterization of this model identified a pathway driven by telomere DNA damage-mediated activation of the ATM/cABL/YAP1 pathway, which directly regulates genes central to IBD pathogenesis and amenable to therapeutic intervention. This review summarizes the evidence correlating telomere dysfunction with IBD and colitis-associated cancer and proposes therapeutic opportunities for such inflammatory conditions targeting this newly identified pathway.

## Introduction

Inflammatory bowel disease, mainly comprised of Crohn’s disease and ulcerative colitis, is a challenging condition associated with both genetic and non-genetic drivers. The mechanisms that drive IBD are not well understood, making it difficult to identify therapeutic targets of pathogenetic relevance [1]. In course of studying the impact of telomere dysfunction on the gastrointestinal system, we identified an unanticipated link between telomere dysfunction and the development of IBD [2].

## About the study

Telomeres are nucleoprotein structures at the ends of chromosomes that protect and maintain chromosomal integrity together with the shelterin protein complex. During cell division, chromosomes must be replicated; however, the replication machinery cannot fully copy to the very end of linear human chromosomes. Telomerase Reverse Transcriptase (TERT) and its RNA component (TERC) act in a coordinate manner to elongate telomeres to solve the end replication problem, where TERC provides the RNA template for telomere repeat synthesis [3]. Genetic diseases associated with short telomeres, termed ‘telomeropathies’ including dyskeratosis congenita, idiopathic pulmonary fibrosis, liver cirrhosis [4] and others have been associated with inflammation for decades. Recently, cumulative evidence from studies on patients with pancreatitis, liver cirrhosis [5], and Inflammatory Bowel Disease (IBD) have established significant telomere shortening in the epithelium with or without genetic polymorphisms in telomere-associated genes [6–8]. Findings of short telomeres in the epithelial cells of IBD patients is particularly interesting given that the patients with telomeropathies exhibited significant inflammation of the gastrointestinal tract that is characterized by cryptitis, sub-mucosal inflammation, villous atrophy, and intra-epithelial lymphocytosis all indicative of severe inflammatory reactions and recruitment of immune cells to the lamina propria. These pathologic findings mirror several features of IBD and bear similarities to the inflammatory phenotype of the telomere dysfunctional mouse model [2]. Despite such strong correlation between telomere dysfunction and inflammation, insights into underlying mechanisms are lacking and could help to identify therapeutic avenues for such obdurate diseases.

A systematic phenotypic and histological analysis of the telomere dysfunctional mouse model (LSL-mTERT; Lgr5-CreERT2) revealed a significant inflammatory phenotype specific to the large intestine with remarkably negligible effect on the small intestine [2].

**Figure 1.**
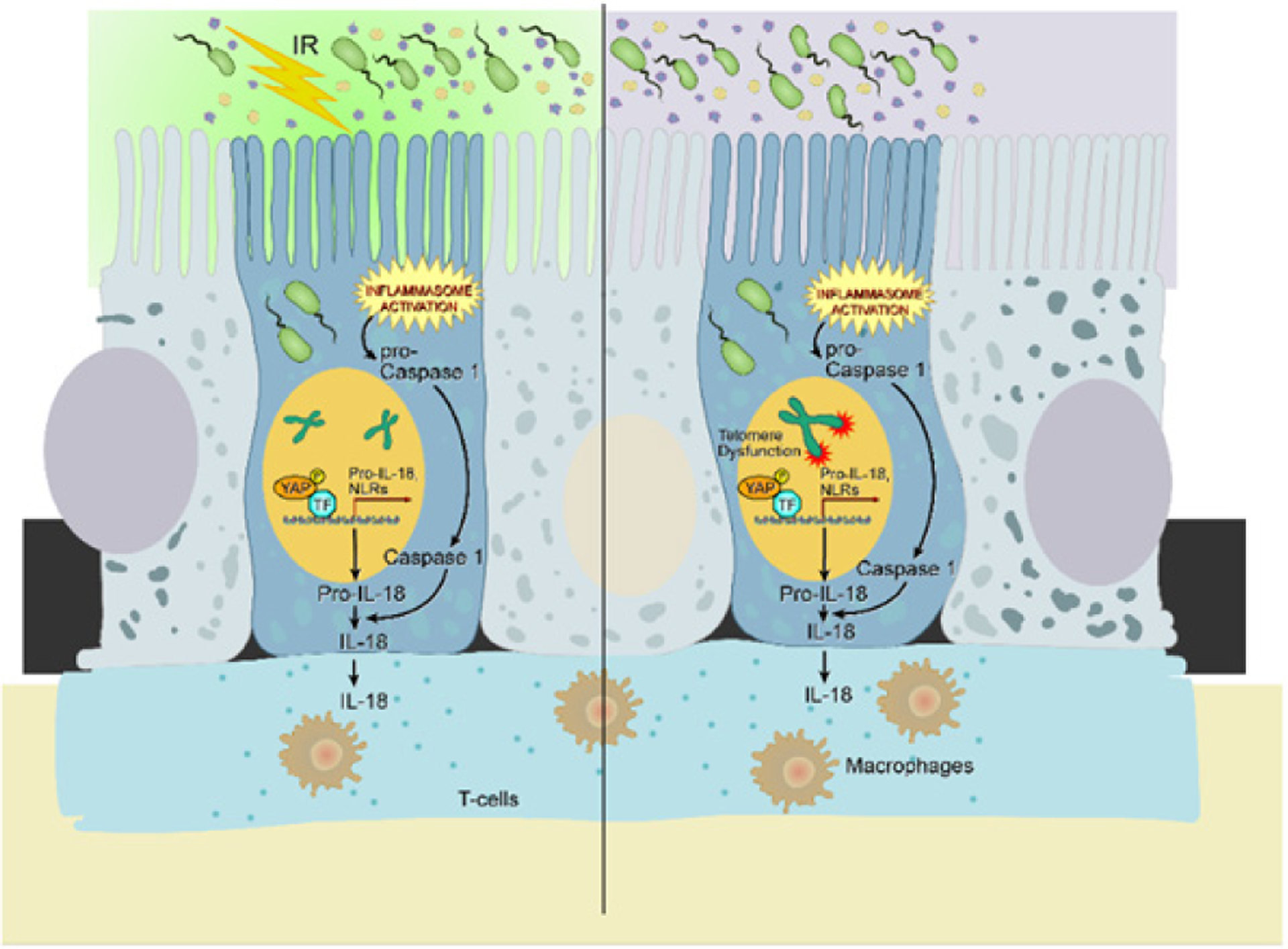
Telomere dysfunction/irradiation initiates intestinal inflammatory cascade through DNA damage mediated activation of the ATM/cABL/pYAP/IL18 axis. IR denotes irradiation. ATM, ataxia telangiectasia mutated; YAP1, Yes associated protein 1; TF, transcription factor; pro-IL18, pro- interleukin 18; cABL, cellular Abelson leukemia virus.

Reactivation of telomerase specifically in intestinal stem cells marked by Lgr5 led to a significant amelioration of inflammation and improvement in colon health. This was also associated with increase in body weight and a significant extension in life-span of these mice, suggesting an overall improvement in fitness. Microscopic and flow-cytometric analyses ascertained an increased recruitment of primarily T cells and macrophages in the lamina propria of the telomere dysfunctional mice and an absence of such immune infiltration in the telomerase reactivated cohorts indicating a direct effect of telomerase reactivation on immune cell recruitment. Transcriptomic analysis of enriched crypt epithelial cells or sorted Lgr5+cells from dysfunctional mice with or without telomerase documented a preponderance of inflammatory pathways in the telomere dysfunctional group compared to the telomerase proficient group. Strikingly, one of the top pathways was the inflammasome pathway, which included several NLR genes as well as the well-studied gene pro-IL18 known for its role in inflammatory bowel disease. Associated with this was also an increase in cleaved caspase-1 further indicating increased cleavage of pro-IL18 to mature IL18. Interrogation of transcriptomic data and *in silico* analysis of the promoter for pro-IL18 revealed various transcription factor binding sites. In particular YAP1 caught our attention given a recent report on a SNP associated with TEF binding element in IBD patient epithelium [2]. Unbiased ChIP-sequencing analysis for YAP1 binding sites on these genes as well as individual validation of YAP1 binding to the promoter site of pro-IL18 revealed strong binding of YAP1 to the promoter, which was reduced on telomerase activation highlighting the role of YAP1 in the regulation of IL18 protein levels Interestingly YAP1 was also found to occupy promoter regions of several NLRs. DNA damage mediated ATM activation and cABL phosphorylation have been shown to phosphorylate YAP1 at tyrosine residue 357, stabilize the protein and localize it to the nucleus. Validation of this pathway in isolated organoids from telomere dysfunctional mice demonstrated high DNA damage, activation of this axis and increased pro-IL18, while reactivation of telomerase in matched organoids and mice revealed a reduction in DNA damage and a corresponding reduction in the pro-IL18 levels. Given that we observed increased transcription of NLR proteins, caspase-1 cleavage and increased mature IL18 levels coupled with the localizationof inflammation in the colon, we reasoned that the gut microbiome may be a cooperating factor in inducing such an inflammatory response. *In vivo* treatment of telomere dysfunctional mice with inhibitors for YAP1 (verteporfin) or caspase-1 (Ac-YVAD) or with broad spectrum antibiotics (trimethoprim-sulfa) ameliorated disease burden and suppressed the ATM/YAP1/IL18 axis revealing cooperation between the two axes telomere dysfunction mediated DNA damage and the microbiome activated inflammasome pathway in driving inflammatory response and highlighting YAP1 as a common orchestrator. Interestingly, we found that DNA damage (irradiation) could also activate this pathway. This study also establishes the important role of intestinal epithelial cells in driving such diseases that may cooperate with the immune cells in exacerbating the disease and driving relapse. To summarize, telomere dysfunction can initiate an inflammatory response through DNA damage mediated activation of the YAP1/pro-IL18 axis in the presence of the gut microbiome (Figure 1).

Chronic inflammation is a driver of various carcinomas [9]. In fact, an estimated 35% of epithelial cancers are driven by chronic inflammation. Not surprisingly, IBD patients are predisposed to developing colorectal cancers at a higher rate [6,10–12]. Cancer development is associated with the duration of ulcerative colitis, with a cumulative risk of 2%, 8% and 18% after 10, 20 and 30 years, respectively [10,13]. In patients with IBD, telomere attrition was shown to be a hallmark feature, especially associated with ulcerative colitis [6,12], raising the possibility that telomere dysfunction in this context might be driving chromosomal instability and cancer progression. However, the sequence of events remains unresolved given that increased inflammation may also lead to telomere shortening and genomic instability [14]. Our study unveils an important, previously unidentified inflammatory axis with telomere dysfunction at the point of initiation which may also fuel colitis associated cancers. Other telomere-related mechanisms that create a cancer-promoting environment have been detailed in a recent review [15].

Telomerase reactivation has been shown to suppress inflammation and promote regeneration in several murine models [16,17]. This strategy could be useful in ameliorating inflammation; however, with more than 90% of cancers reactivating telomerase to sustain growth and metastasis [15], caution should be taken in the ideation of such a strategy. Rather than continuous telomerase reactivation, we propose a model of intermittent cycles of telomerase reactivation accompanied by aggressive monitoring for loss of cell cycle check point genes like p53 and RB, given that these genes are associated with telomere dysfunction and carcinoma progression [15–19]. Other noteworthy therapeutic strategies would include the suppression of YAP1 or its downstream targets including IL18.

## Conclusion

In support of this notion, IL18 has been proposed as a viable target in Mendelian enterocolitis, and an anti-IL18 antibody has been found to be safe in a Phase II trial of diabetes, albeit without efficacy. This could serve as an excellent opportunity for drug repurposing. Lastly, the ATM/YAP1/IL18 axis could also serve as a key pathway in driving other inflammatory diseases such as Idiopathic Pulmonary Fibrosis (IPF), a condition known to be associated with telomere dysfunction and aid in identifying viable therapeutic targets for such chronic conditions with limited therapeutic options.
